# Platinum Nanoparticles: The Potential Antioxidant in the Human Lung Cancer Cells

**DOI:** 10.3390/antiox11050986

**Published:** 2022-05-18

**Authors:** Noor Akmal Shareela Ismail, Jun Xin Lee, Fatimah Yusof

**Affiliations:** Biochemistry Department, Faculty of Medicine, Universiti Kebangsaan Malaysia, Jalan Yaacob Latif, Bandar Tun Razak, Kuala Lumpur 56000, Malaysia; a161478@siswa.ukm.edu.my (J.X.L.); p74488@siswa.ukm.edu.my (F.Y.)

**Keywords:** platinum nanoparticles, lung cancer, A549 epithelial lung cell, antioxidant, oxidative stress, reactive oxygen species

## Abstract

Oxidative stress-related conditions associated with lung cells, specifically lung cancer, often lead to a poor prognosis. We hypothesized that platinum nanoparticles (PtNPs) can play a role in reversing oxidative stress in human lung adenocarcinoma A549 epithelial lung cell lines. Hydrogen peroxide (H_2_O_2_) was used to induce oxidative stress in cells, and the ability of PtNPs to lower the oxidative stress in the H_2_O_2_ treated epithelial lung cell line was determined. The differential capacity of PtNPs to remove H_2_O_2_ was studied through cell viability, nanoparticle uptake, DNA damage, ROS production, and antioxidant enzymes (superoxide dismutase, glutathione peroxidase, and catalase). Results indicated that a higher concentration of PtNPs exhibited a higher antioxidant capacity and was able to reduce DNA damage and quench ROS production in the presence of 350 µM H_2_O_2_. All antioxidant enzymes’ activities also increased in the PtNPs treatment. Our data suggested that PtNPs could be a promising antioxidant in the treatment of lung cancer.

## 1. Introduction

The balance between oxidant and antioxidant defense mechanisms is important to maintain the metabolic state of normal cells. Oxidative stress conditions or diseases are the endpoints of the imbalance between these mechanisms [1]. This is due to changes in the structure of proteins and nucleic acids and an increased permeability of the membrane to injury and lipid peroxidation [1]. The accumulation of reactive oxygen species (ROS) takes place and subsequently induces cellular oxidative damage. Reactive oxygen species (ROS) are highly reactive molecules generated from the oxidation process when molecular oxygen (O_2_) is reduced to produce superoxide (O_2_^•−^), which is the precursor to most other reactive oxygen species [2]. Cells that undergo oxidative stress are unable to function normally and ROS will attack various cell components including lipids, enzymes, and DNA [3]. Some organs will prevent oxidative damage through enzymatic and non-enzymatic antioxidant defense mechanisms [4]. Enzymatic antioxidant defense mechanisms consist of superoxide dismutase (SOD), glutathione peroxidase (GPx), and catalase (CAT), while non-enzymatic antioxidant defense mechanisms originate from various sources such as vitamin C, vitamin E, and uric acid [5].

Lung cancer remains one of the most common types of cancer and the leading cause of cancer-related deaths worldwide despite considerable advancements in diagnostics and management [6]. Oxidative stress in lung cells can be a deleterious process that leads to lung cancer that carries a poor prognosis. It plays a crucial role in carcinogenesis by promoting tumor growth and inducing neoplastic transformation [7]. While exogenous antioxidants through daily diet consumption and derivatives of medicinal plants have been intensively explored as potential preventive agents [8], the role of endogenous antioxidants in lung cancer is ambiguous as various antioxidant enzymes have been found to promote neoplastic cell viability [9]. Hence, ROS regulation is one of the essential fields of study, especially in the presence of antioxidant agents as a pharmacological manipulation in lung inflammation and injury.

The use of cisplatin and platinum-based chemotherapy has been the fundamental treatment of lung cancer, but it has also been shown to cause organ toxicities, especially in the kidney, gastrointestinal, and neuron cells [10]. These adverse events can interfere with the treatment of lung cancer. Therefore, the elucidation of its mechanism to treat lung cells is important to explore the potential of platinum nanoparticles (PtNPs) in reducing cell toxicity. Metal nanoparticles have raised considerable interest in research due to their material properties, availability, capabilities, specific targeting, and sustainable release [11]. PtNPs specifically are chosen as an antioxidant candidate in this study as PtNPs can reverse antioxidant activity in several induced oxidative conditions [12,13,14,15] and have quenched the production of free radicals, thus reducing the impact of oxidative damage [16,17]. PtNPs have garnered attention in medicine due to their reactivity to cells and are renowned for their therapeutic application in different cell lines. PtNPs have successfully induced cell death in cervical cancer cells [18], breast cancer cells [19], and human colon carcinoma cell lines HT29 [20,21], notified by reduced DNA damage and antioxidant response. In addition, PtNPs do not show a cytotoxicity effect in several established cultured cells (TIG-1, WI-38, MRC-5, HeLa, and HepG2) whilst the cellular uptake of PtNPs does occur in a time- and dose-dependent manner [22]. The exposure of PtNPs through in vivo experiments has been found to only affect lung cells but not the brain, the kidney, the heart, and liver cells [13]. It was first discovered as an antioxidant through the ability to protect the inflamed lung cells from further oxidation-induced inflammation by scavenging superoxide anions (O_2_^•−^) and hydroxyl radicals (OH^•^) from aqueous solutions [13].

Thus, this study aimed to determine the antioxidant properties of PtNPs in balancing oxidative status in human lung adenocarcinoma A549 epithelial lung cell line as an in vitro model; an area in which the available evidence is scarce. As such, the tests that were being carried out included the Ferric Reducing Antioxidant Power (FRAP) assay, the MTS assay, the comet assay, and antioxidant enzymes (superoxide dismutase, glutathione peroxidase, catalase) activity to further elucidate the antioxidative role of PtNPs.

## 2. Materials and Methods

### 2.1. Platinum Nanoparticles and Hydrogen Peroxide

Platinum nanoparticles (PtNPs) colloids were purchased from Sigma-Aldrich, St. Louis, MI, USA. The PtNPs were 3 nm in size, without any prior modification or purification done. This is according to a study made by the previous literature that suggested a similar size of diameter of each PAA-Pt nanoparticle (2.0 ± 0.4 nm) as these PtNPs exhibit the chemical composition, biological reactivity, and antioxidant properties of the nanoparticles [13]. Hydrogen peroxide (H_2_O_2_) was purchased from Merck, Germany. This was to induce an oxidative condition in the cell line.

### 2.2. Treatment Procedure

A549 cells were cultured between passages P17 to P25 that were at the growth phase. Six treatment groups were used during this study that were the untreated group (control), cells with PtNPs and H_2_O_2_, respectively, and H_2_O_2_/PtNPs treatment. There were two durations, 3 h representing the acute treatment, and 24 h for the chronic treatment. The number of cells used per well is 0.5 × 10^6^ for a petri dish and 5 × 10^3^ for each of the 96-well.

### 2.3. Cell Culture

Human lung adenocarcinoma epithelial cell line A549 was purchased from American Type Culture Collection (ATCC), item number CCL-185. A549 cells were cultured in Dulbecco’s Modified Eagle’s Medium-F12 (DMEM-F12), with additional _L_-glutamine supplemented with 10% Fetal Bovine Serum (FBS), 1% penicillin/streptomycin, and 1% amphotericin B. Cells were incubated in a humidified atmosphere at 37 °C in 5% CO_2_. All chemicals for cell culture were obtained from Biowest, USA.

### 2.4. Ferric Reducing Antioxidant Power (FRAP) Assay

FRAP assay was carried out to study the antioxidant capacity, based on the ability of the sample to reduce Fe^3+^ to Fe^2+^. FRAP reagent was freshly prepared by mixing acetate buffer, FeCl_3_·6H_2_O solution, and 2,4,6-Tris(2-pyridyl)-s-triazine (TPTZ) in a 10:1:1 ratio [23]. The concentration of PtNPs stock used was 1000 µg/mL and the concentration of ascorbic acid (Sigma-Aldrich) stock was 5000 µg/mL. Ascorbic acid acts as a positive control for antioxidant properties. Absorbance readings were taken at 593 nm using a microplate reader (PerkinElmer, Waltham, MA, USA). The antioxidant capacity of the sample was determined by plotting a standard curve of FeSO_4_·7H_2_O.

### 2.5. MTS Assay

This colorimetric assay was used to determine the number of living cells through the how much NAD(P)H-dependent cellular oxidoreductase enzymes convert 3-(4,5-dimethylthiazol-2-yl)-5-(3-carboxymethoxyphenyl)-2-(4-sulfophenyl)-2H-tetrazolium (MTS) (Invitrogen, Paisley, UK) to formazan and is soluble in aqueous media cell culture [24]. Thus, the number of living cells can be measured directly by measuring the quantity of formazan produced at an absorbance of 490 nm. After treatment duration, cells were washed with phosphate-buffered saline (PBS). The mixture of 20 µL MTS solution and 100 µL media was added to each well for 2 h. Cell viability percentage was calculated based on the ratio of mean optical density from each treatment group to the mean OD control group.

### 2.6. Nanoparticles Uptake

This protocol is adapted from the processing tissue and cells for transmission electron microscopy protocol [25]. A549 cells were treated with 100 µg/mL PtNPs for 3 and 24 h. After the treatment duration, cells were washed with PBS three times. Next, the cells were trypsinized and washed with PBS again to get rid of unbound PtNPs. Cells were fixed with 3% glutaraldehyde for 30 min and underwent the second fixation with 1% osmium tetroxide for 20 min. Cells were washed with PBS before being blocked by 0.5–3.0% uranyl acetate for an hour. Serial ethanol percentage was carried out before infiltration with resin. Cells were embedded in a 100% resin capsule and hardened at 70˚C. Subsequently, cells were cut ultrathin and stained with uranyl acetate. Cells in grid form were analyzed using a transmission electron microscope (Philips, Hillsboro, OR, USA).

### 2.7. Comet Assay

DNA damage in A549 cells was measured by the comet assay based on the protocol by Olive and Banáth, 2006 [26]. Cells were embedded in agarose and lysed by lysis solution in a Coplin jar. Electrophoresis was done in an alkaline solution to separate DNA in the nucleus. DNA damage formed a comet-like tail due to DNA fragments that move faster than the nucleus. After undergoing the neutralization process, cells were stained with Ethidium Bromide (EtBr). Cells with intact nucleus appeared as intact orange dots under a fluorescence microscope (Carl Zeiss, Jena, Germany). A total of 500 cells that did not overlap were selected randomly from each of the slides. Grading was done on each cell according to a scale of 0 to 4 based on DNA damage level. The total cell count for each grade was multiplied by the number of each grade and summed together for total DNA damage.

### 2.8. Reactive Oxygen Species (ROS) Production

2′,7′-dichlorodihydrofluorescein diacetate (H_2_DCF-DA) and dihydroethidium (DHE) were used to detect ROS production. DHE produced red fluorescence when oxidized by superoxide radicals while H_2_DCFDA emitted green fluorescence when oxidized by H_2_O_2_. After treatment, cells were washed with 50 mM PBS before staining with either 5 mM DHE or 50 mM H_2_DCFDA. Plates were incubated for 30 min. Then, cells were washed again to remove the excess dye in the cell before the media was added to each well for 30 min. Next, cells were washed before being trypsinized using 500 µL trypsin. The cell suspension was added to 800 µL of 50 mM PBS before being transferred into 1.5 mL centrifuge tubes and spun at 3000× *g* for 5 min. The supernatant was removed, and the pellet was dissolved in 200 µL of 50 mM PBS. Changes in the fluorescence intensity of the cell population to the right side of the *X*-axis than in the control group showed ROS production increased. A total of 10,000 cells were used for each sample and results were obtained using a flow cytometer (BD Facsverse). This protocol was adapted from Wojtala et al. [27].

### 2.9. Antioxidant Enzymes

Superoxide dismutase (SOD) and glutathione peroxidase (GPx) activity were measured using the commercially available kit (Cayman Chemicals, USA) following the protocol provided by the manufacturer. Catalase (CAT) enzyme activity was determined by using 50 mM PBS buffer, pH 7.0, based on a protocol by Aebi et al. [28]. The protein concentration of each sample was determined in advance using the Bradford reagent. During the experiment, 1.5 ug/mL of extract enzymes were added to each well. Control wells were added to with 50 mM PBS buffer meanwhile 0.01 M H_2_O_2_ was inserted into the sample wells. Absorbance readings were recorded at 240 nm at 0 and 30 s.

### 2.10. Statistical Analysis

All data were analyzed as mean ± standard deviation for the value obtained at least in three independent experiments. The data were analyzed using the one-way analysis of variance (ANOVA) by SPSS version 22. *p*-values of less than 0.05 (*p* < 0.05) were accepted as significant results and denoted as a comparison between two groups in an alphabet.

## 3. Results

### 3.1. Antioxidant Capacity of PtNPs was Lower Than Ascorbic Acid

**Figure 1:**
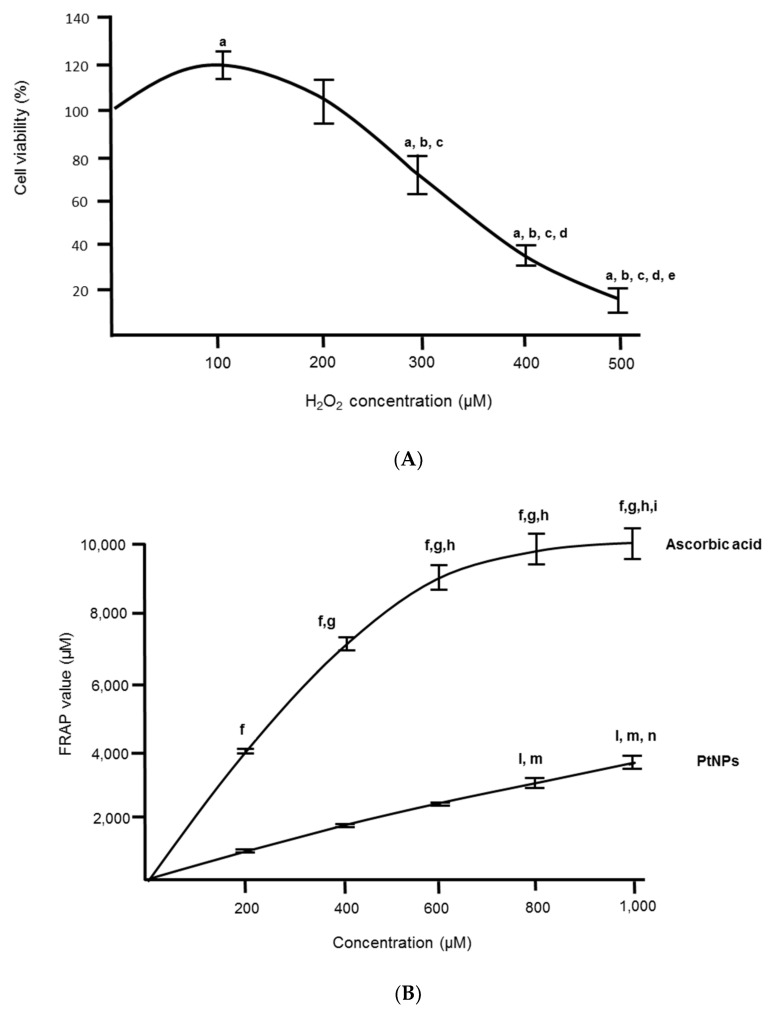
(**A**) Cell viability percentage of H_2_O_2_ concentration (n = 3). A statistically significant difference (*p* < 0.05) denoted in alphabet a–e represents a comparison between a group to, a: untreated H_2_O_2_ (0 µM), b: 100 µM, c: 200 µM, d: 300 µM, and e: 400 µM. (**B**) antioxidant capacity of PtNPs and ascorbic acid using FRAP assay (n = 3). A statistically significant difference (*p* < 0.05) denoted in alphabet f–i (ascorbic acid) and l–n(PtNPs) that represents a comparison between a group to, f and l: untreated cells (0 µg/mL), g and m: 200 µg/mL, h and n: 400 µg/mL, i: 600 µg/mL.

An antioxidant capacity of PtNPs was first initiated to determine the optimal dosage prior to subsequent experiments. The half-maximal inhibitory concentration (IC50) has been determined through the half percentage of the cell viability in the oxidation of H_2_O_2_ at 350 µM (Figure 1A). The percentage of cell viability reduces when the duration of different PtNPs concentration treatments is increased. PtNPs treatment groups that are pre-treated with H_2_O_2_ also show a lower percentage of cell viability in prolonged treatment (Figure 1B).

Next, the antioxidant capacity of the PtNPs that was measured with the FRAP assay was significantly lower than ascorbic acid (Figure 1B). PtNPs were able to convert ferric ions to ferrous ions lower than in ascorbic acid. The scavenging of free radical activity of PtNPs was still durable even though the reading of the FRAP value in PtNPs was much lower than ascorbic acid. The amount of PtNPs in the FRAP assay can only be measured up until 1000 µg/mL as that was the highest concentration in the initial stock. Ascorbic acid (AA) shows higher antioxidant capacity at all concentrations than platinum nanoparticles (PtNPs).

### 3.2. Cell Viability Was Better in Acute PtNPs

**Figure 2:**
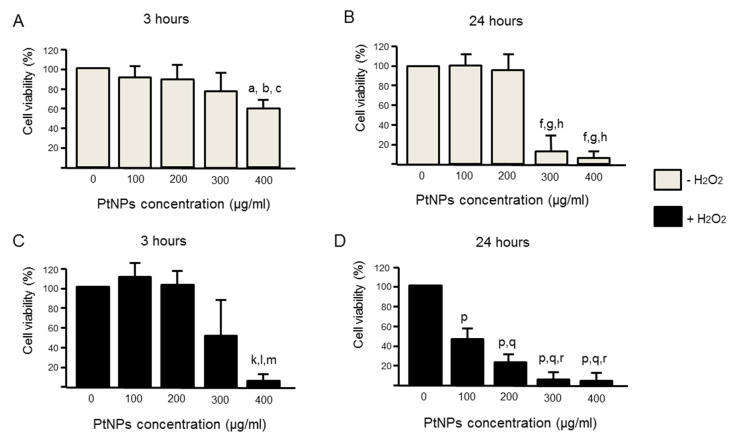
Cell viability in different PtNPs concentrations in two different time points (n = 3), (**A**) 3 h and (**B**) 24 h are measured by determining antioxidant capacity using FRAP assay. The 50% cell viability is determined at 400 ug/mL for 3 h and in 24 h, 300 and 400 ug/mL have shown minimal cell viability as compared to other dosages. Subsequently, several concentrations of PtNPs were introduced after cells were pretreated with H_2_O_2_ in two different durations, (**C**) 3 h and (**D**) 24 h. A statistically significant difference (*p* < 0.05) denoted in alphabet a–c (**A**), f–h (**B**), k–m (**C**), and p–r (**D**) that represents a comparison between a group to, a/f/k/p: untreated H_2_O_2_ (0 µg/mL), b/g/l/q: 100 µg/mL, c/h/m/r: 200 µg/mL.

The half-maximal inhibitory concentration (IC50) had shown the half percentage of the cell viability through the oxidation of H_2_O_2_ was at 350 µM (Figure 1A). Based on this data, this dosage was used in all experiments. We had chosen two different durations, acute (3 h) and chronic (24 h) exposure of PtNPs. A longer incubation of PtNPs revealed less cell viability (Figure 2B) as compared to 3 h (Figure 2A). In the acute treatment (3 h), 100 ug/mL showed the highest cell viability where the 50% cell viability is noted at 300 ug/mL (Figure 2C). In the prolonged treatment (24 h), all ranges of concentration used in this experiment have shown low cell viability (Figure 2D). When PtNPs were introduced in an oxidized condition (pre-treated with H_2_O_2_), PtNPs can reverse the effect caused by the oxidant in 100 µg/mL (Figure 2C).

### 3.3. The Level of Antioxidant Enzymes was Elevated in the Presence of PtNPs

**Figure 3:**
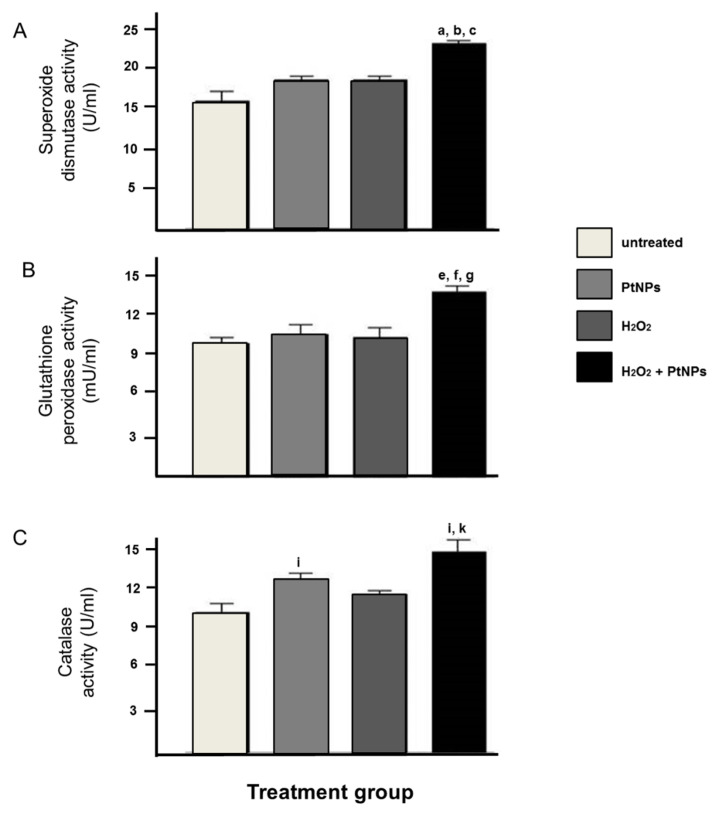
Cells were treated with H_2_O_2_ and PtNPs for 3 h and subjected to the level of antioxidants activity. PtNPs were shown to increase the activity of antioxidants enzymes of (**A**) superoxide dismutase, (**B**) glutathione peroxidase, and (**C**) catalase activities. A statistically significant difference (*p* < 0.05) denoted in alphabet a–c (**A**), e–g (**B**), and i, k (**C**) that represents a comparison between a group to, a/e/i: untreated cells, b/f: PtNPs, and c/g/k: H_2_O_2_.

For the analysis of the level of antioxidant enzymes, the PtNPs treatment had significantly increased the antioxidant activity of SOD, GPx, and CAT in pre-treated H_2_O_2_ cells when compared to the H_2_O_2_ treatment group (Figure 3A–C).

### 3.4. PtNPs Uptake was Seen in the Nucleus

**Figure 4:**
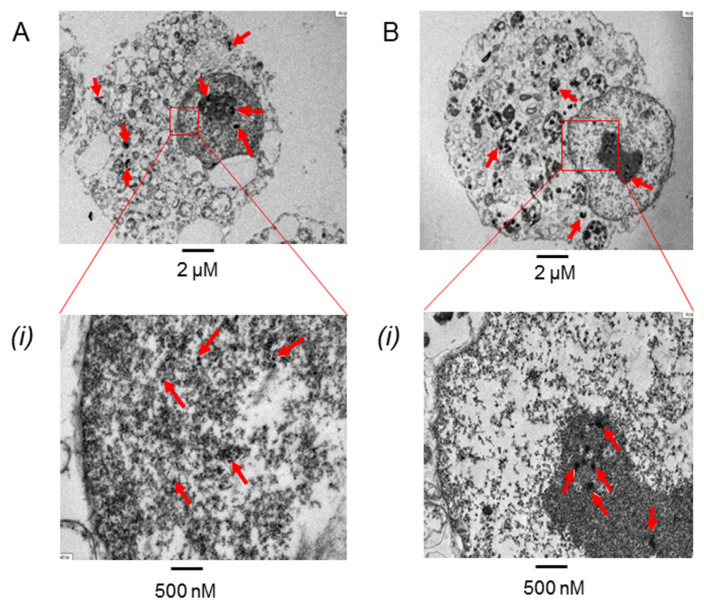
TEM image of PtNPs uptake by A549 cells (**A**) 3 h and (**B**) 24 h treatment (Scale bar: whole cell, 2 nm). The scale was enlarged to focus on the nucleus (500 µm). Red arrows indicate the accumulation of PtNPs. PtNPs can penetrate the cells as early as 3 h however the cells keep accumulating in the cytoplasm and nucleus in prolonged treatment (24 h).

This study was conducted using 3 nm PtNPs without any surface protectant during PtNPs synthesis. We aimed to show the direct effect of PtNPs on the cells. Indeed, we found that PtNPs can penetrate the epithelial cell in as early as 3 h of treatment and can enter the nucleus membrane in both treatments (Figure 4A). However, 24 h of treatment allowed more time for PtNPs to accumulate in the cytoplasm and nucleus (Figure 4B).

### 3.5. PtNPs Can Reduce Oxidative Condition in H_2_O_2_ Treated Cells

**Figure 5:**
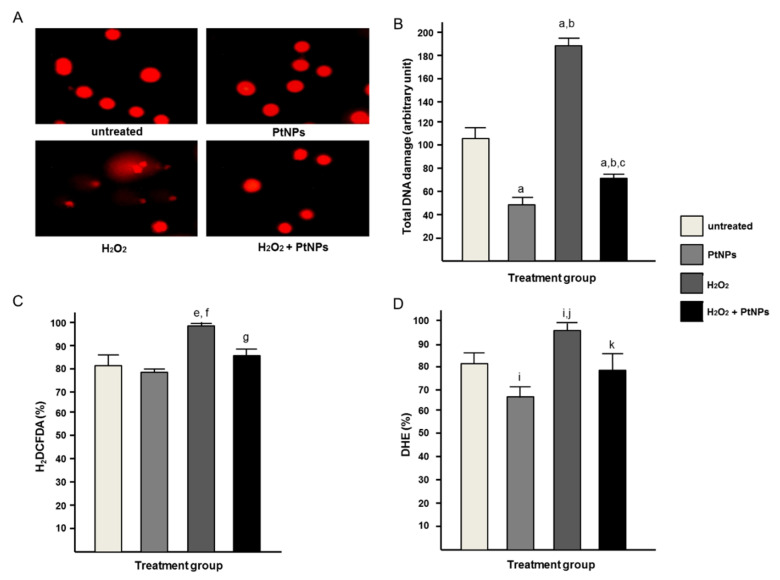
(**A**) Oxidative status through DNA damage represented with comet assay qualitatively. Comet tail can be seen in DNA damaged cells and quantitatively measured in (**B**) total DNA damage and ROS production represented (**C**) H_2_DCFDA and (**D**) DHE, respectively. A statistically significant difference (*p* < 0.05) denotes in alphabet a–c (**A**), e–g(**B**), i–k (**C**) that represents a comparison between a group to a/e/i: untreated cells, b/f/j: PtNPs, and c/g/k: H_2_O_2_.

DNA damage can be seen clearly in the H_2_O_2_ treatment group, but this damage is reduced significantly both qualitatively (Figure 5A) and quantitatively (Figure 5B) in the presence of PtNPs. The PtNPs treatment group showed significantly lower DNA damage as compared to the control group. The production of ROS was significantly reduced by the H_2_O_2_/PtNPs treatment group as compared to the H_2_O_2_ treatment group. In parallel with DNA damage data, the PtNPs treatment group also showed a significant reduction in H_2_DCFDA (Figure 5C) and DHE (Figure 5D) compared to the control group.

## 4. Discussion

Platinum-based compounds, mainly cisplatin and carboplatin, have been the most commonly used chemotherapeutic agents in the practice of lung cancer treatment. Nevertheless, there are several profound complications, especially neuro- and nephrotoxicity [10], whilst the response and survival rate among lung cancer patients are still poor. Hence, our study studied the prospective role of PtNPs against lung cancer through an in vitro model to see its antioxidative effect in an oxidant condition. Our study revealed that PtNPs have exhibited antioxidant properties by scavenging free radicals induced by hydrogen peroxide. Whilst its antioxidant capacity is relatively lower than vitamin C, results from SOD, GPx, and CAT were significantly increased when compared to the untreated control group. This is parallel to the first finding suggesting that PtNPs exhibit antioxidant properties in reducing oxidative damage in mouse lung models induced by inflammation [13]. This data is also in parallel to the previous study where they found a similar reduction in antioxidant status but with a different lung cell line model [29,30]. Besides, our study also revealed a significant reduction in ROS production with PtNPs treatment that was similar to other studies worldwide [16,17]. Among all metal nanoparticles, PtNPs were seen to be the strongest quencher of reactive oxygen species, namely hydrogen peroxide and superoxide anion, and able to act as SOD mimetics in a dose-dependent manner [16] whilst another study also demonstrated the capability of PtNPs in inhibiting hyperthermia-induced apoptosis in lymphoma cells [31]. The superoxide dismutase and catalase activities exhibited by PtNPs have also been shown in studies to further potentiate its interventional effect in aging-related skin [12], skeletal muscle [32], vascular endothelium [33], and monocytic leukemia [34] in vitro models. This is evident by our study that also revealed that PtNPs had significantly increased the antioxidant activity of SOD, GPx, and CAT in the A549 cell line in supporting the capability of PtNPs as antioxidants, as established in other studies [35,36,37,38,39]. In comparison to different metal nanoparticles that exhibit antioxidant activities, platinum nanoparticles are among one the best antioxidants to increase all CAT, GPx, and SOD [39]. PtNPs were also proposed to be a catalase (CAT) mimicker and have been used to enhance radiation efficacy in the treatment of cancer [38,39]. PtNPs are a suitable candidate as the smaller size of PtNPs produces a lower oxidation of H_2_DCFDA compared with the bigger size due to the larger surface area to enhance the catalytic activity [29]. Looking at numerous data that support PtNPs as a potential antioxidant, it is worth to further scrutinize the mechanisms involved in between exerting the antioxidant properties and inducing apoptosis. This could be due to the blockage in different stages of the cell cycle, changes to the cell membrane, disruption and alteration in the cell homeostasis, and enzyme activity. Hence, it is worth to subsequently perform cell cycle analysis and establish the mechanism of cell death in various concentrations of PtNPs and exposures.

The unique features of PtNPs, such as surface functionalities; size; size distribution; shape; porosity; surface area; composition; crystalline nature; agglomeration; and electro, catalytic, thermal, and plasmonic properties, making their application desirable in various fields [40,41]. The capping of nanoparticles with stabilizing agents aims to prevent aggregation while in liquid suspension. However, the porous organic capping agents have open spaces that allow reactant and product molecules to reach the metal catalysts, causing a reduction in catalytic activity as certain elements in the capping agents can block the reactive sites on nanoparticles [42]. Hence, the uncapping of PtNPs was used throughout this study to ensure better penetration to the nucleus and to observe its antioxidant capacity, which fits the main aim of our study. A study was also conducted on pure PtNPs in vitro, which showed that there was no release of metal ions into the media [43]. This suggests that unmodified PtNPs do not contribute to cytotoxicity based on metal ion release. Even though different sizes of PtNPs (5–10 nm) can penetrate A549 and HaCaT cells within 24 h of treatment, these nanoparticles were not found to be present in the nucleus in acute treatments [43], which coincides with our data.

The efficacy of PtNPs treatment as an antioxidant is strongly dependent on the type of cell, size, concentration, and duration of care settings. The size of PtNPs used in this study is 3 nM, which was relatively small, therefore, cytotoxicity could be caused by prolonged exposure as it can penetrate to the cell membrane of epithelial lung cancer cells over time. In fact, an in vitro study has shown that 1 nm PtNPs are capable of reducing superoxide anion radicals better than 5 nm PtNPs [22]. Furthermore, our data supported that the cells did not survive well with chronic exposure to PtNPs by keeping the PtNPs longer than 3 h. There were similar findings in other studies; notably, smaller sizes (<100 nm) of PtNPs can penetrate directly into the cell when on the other hand, larger sizes (>100 nm) must be taken in through conventional endocytosis [43], which could lead to a higher cytotoxicity toward the cells.

With the chronic exposure in a longer duration, PtNPs were seen to have accumulated in both the cytoplasm and the nucleus, which could lead to less antioxidative effects. The coagulation and sedimentation of the nanoparticles may limit the antioxidant activity due to the instability of the nanoparticles in the medium. Nanoparticles that tend to clump may disrupt the function of organelles within the cell. The addition of FBS and human serum albumin (HSA) can inhibit the clotting process thus improving the stability of the nanoparticles [44], and this can be a suggestion to further ameliorate the effect of PtNPs as an antioxidative agent in in vitro models. Studies have revealed that higher dosages of PtNPs, and prolonged (48 h) exposure in the A549 lung cell line can cause less cell viability [45], and a shorter exposure duration was able to decrease nanotoxicity to the cells [39]. This data could serve as a great starting point for several treatments in quenching ROS, especially in the chemodynamic therapy, synergistic therapy, and the controlled drug release of nanomaterials [46].

This study has its own limitation in the route to decipher the antioxidative role of PtNPs. Since the sizes of PtNPs are small, chronic exposure could lead to DNA damage and surpass the beneficial role of PtNPs. Hence, a study needs to be conducted to further compare different agents in the capping of PtNPs using various amphiphilic molecules comprised of a polar head group and a non-polar hydrocarbon tail [47,48] to further stabilize the nanoparticle and specifically target the cancer cells [49,50]. This might include polyethylene glycol (PEG) [51], polyvinylpyrrolidone (PVP) [40], polycaprolactone (PCL) [52], chitosan [53], and plant extracts [15,54,55,56]. All of these capping agents are capable of further alleviating cellular toxicity [57] through minimal agglomeration of the nanoparticles [58], especially in the nucleus.

## 5. Conclusions

The present study has shown the antioxidant property of PtNPs in reversing oxidative stress conditions in epithelial lung cancer in an in vitro model. Despite the low antioxidative effect shown by the PtNPs, the nanoparticles are capable of reversing the oxidative stress status at a lower concentration. The small size of PtNPs exhibited better penetration of the nucleus and was able to reduce DNA damage and ROS production when cells were under oxidative stress. Hence, we recommend the potential of PtNPs as an antioxidant in reducing oxidative stress, subjected to further testing on different cell lines and in vivo models. This antioxidative potential should be observed in a low concentration of PtNPs to ensure a non-toxic environment for the cells. Modification of PtNPs, especially through encapsulation, can reduce the reaction time and energy required and offer ambient conditions of fabrication that would be another promising way forward in the treatment of lung cancer.

## Data Availability

Data is contained within the article.
